# Prognostic Value of *KRAS* Mutations in Colorectal Cancer Patients

**DOI:** 10.3390/cancers14143320

**Published:** 2022-07-07

**Authors:** Asimina Koulouridi, Michaela Karagianni, Ippokratis Messaritakis, Maria Sfakianaki, Alexandra Voutsina, Maria Trypaki, Maria Bachlitzanaki, Evangelos Koustas, Michalis V. Karamouzis, Anastasios Ntavatzikos, Anna Koumarianou, Nikolaos Androulakis, Dimitrios Mavroudis, Maria Tzardi, John Souglakos

**Affiliations:** 1 Laboratory of Translational Oncology, Medical School, University of Crete, 70013 Heraklion, Greece; asi_minakoulouridi@yahoo.com (A.K.); michaelakaragianni@yahoo.gr (M.K.); mimasf19@gmail.com (M.S.); voutsinalex@gmail.com (A.V.); tr.maria@gmail.com (M.T.); mavroudis@uoc.gr (D.M.); 2 Medical Oncology Unit, Pananio-Venizelio General Hospital of Heraklion, 71500 Heraklion, Greece; mara.bach@hotmail.com (M.B.); nandroulakis@yahoo.gr (N.A.); 3 Molecular Oncology Unit, Department of Biological Chemistry, Medical School, National and Kapodistrian University of Athens, 11527 Athens, Greece; vang.koustas@gmail.com (E.K.); mkaramouz@med.uoa.gr (M.V.K.); 4 Hematology Oncology Unit, Fourth Department of Internal Medicine, Attikon University Hospital, Medical School, National and Kapodistrian University of Athens, 12462 Athens, Greece; msfakianak@uoc.gr (A.N.); akoumari@yahoo.com (A.K.); 5 Department of Medical Oncology, University General Hospital of Heraklion, 70013 Heraklion, Greece; 6 Laboratory of Pathology, Medical School, University of Crete, 70013 Heraklion, Greece; tzardi@med.uoc.gr

**Keywords:** colorectal cancer, *KRAS*, mutations, prognosis

## Abstract

**Simple Summary:**

Kirsten rat sarcoma viral oncogene homolog (*KRAS*)-mutated colorectal cancer (CRC) seems to have a different biological behavior and therapeutic approach compared with non-*KRAS* mutated CRC. Except for the proven predictive significance of *KRAS* mutations in CRC patients, their prognostic significance is still under evaluation. Our study shows that 93.2% of *RAS*-mutated patients were *KRAS* mutants, with G12D being the most common subtype. *KRAS* G12D mutation is correlated with better overall survival (OS). *KRAS* G12C mutations may indicate worse prognosis regarding progression free (PFS) and OS, as well as exon 4 and exon 3 *KRAS* mutations for PFS and OS, respectively. Further studies are warranted to confirm these results.

**Abstract:**

Colorectal cancer (CRC) remains a major public health issue. The detection of parameters that affect CRC prognosis is of great significance. *KRAS* mutations, play a crucial role in tumorigenesis with a strong predictive value. *KRAS*-mutated stage-IV CRC patients gain no benefit of the anti-EGFR therapy. The *KRAS* G12C mutation subtype is under investigation for treatment regimens. The present study aimed to detect various *RAS* mutations in a cohort of 578 *RAS*-mutated CRC patients; 49% of them had de novo metastatic disease; 60% were male; 71.4% had left-sided tumors; and 94.6% had a good performance status. *KRAS* mutations were detected in 93.2% of patients, with *KRAS* G12D being the most common subtype (30.1%). *KRAS* mutations presented shorter progression-free (PFS) and overall survival (OS), compared with *NRAS* mutations, although not significantly (PFS: 13.8 vs. 18.5 months; *p* = 0.552; OS: 53.1 vs. 60.9 months; *p* = 0.249). *KRAS* G12D mutations presented better OS rates (*p* = 0.04). *KRAS* G12C mutation, even though not significantly, presented worse PFS and OS rates. *KRAS* exon 3 and 4 mutations presented different PFS and OS rates, although these were not significant. Concluding, *KRAS* G12D and G12C mutations lead to better and worst prognosis, respectively. Further studies are warranted to validate such findings and their possible therapeutic implication.

## 1. Introduction

Colorectal cancer (CRC) is the third most frequent cancer in the world and the second in mortality rates [1]. In 2020, two million new cases and one million deaths were attributed to CRC, according to the International Agency for Research on Cancer (IARC). Many genetic and environmental factors are responsible for the development of CRC. Amongst them are mutations in the RAS family genes, which are detected in 52% of CRC patients [2]. The *RAS* status in patients with stage IV CRC is critical for the therapeutic decision, as mutation in the *RAS* genes suggests inefficacy of anti-EGFR treatments [2].

The CRC pathway of tumorigenesis that involves *RAS* mutations is chromosomal instability 1 (CIN-1) [3]. The traditional adenoma–carcinoma pathway begins when mutations that inactivate the *APC* tumor suppressor gene occur. Kirsten rat sarcoma viral oncogene homolog (*KRAS*) mutation follows, leading to larger adenomas [3,4]. Finally, additional mutations in *TP53*, *PIK3CA* and loss of 18q lead to invasive cancer [4].

The *RAS* genes are translated into four proteins: *KRAS*-4A, *KRAS*-4B, *HRAS* and *NRAS*. When growth factors, like EGFR, bind to the membrane receptors, RAS proteins transmit the signal to the intracellular space [2], and activate the *RAS/RAF/MAPK* and *PI3K/AKT* pathways, promoting cell proliferation [5]. *KRAS* mutations lead to an always-activated *KRAS* by not binding its inhibitors. As a result, the stimulus for cell growth and proliferation is continuous and carcinogenesis occurs [5]. This is an explanation for the resistance of *KRAS*-mutated CRC to EGFR-targeted therapy [5].

*KRAS* and *NRAS* mutations are seen in about 44.7% and 7.5% of CRC patients, respectively [2]. Amongst *KRAS* mutations, those of exon 2 are the most common, whereas mutations of exon 3 and 4 include 1 to 4% of cases [1]. Furthermore, studies have shown that KRAS-mutated CRCs, more often encountered in males, are adenocarcinomas with well or moderate differentiation and microsatellite stability [2]. Mutation frequency between right (RCC) and left colorectal cancer (LCC) also differs. It has been demonstrated that *KRAS* mutations are more frequently present in RCC than LCC, amongst multiple ethnicities and age groups [6].

*RAS* mutations could be detected in the tissue of both primary and metastatic sites and there is no strongly suggested methodology [2]. Allele-specific PCR, PCR high-resolution melting assays, Sanger sequencing and next-generation sequencing are those most frequently used [2]. Moreover, liquid biopsy can detect RAS mutations in patients with stage IV CRC with great precision [2]. Liquid biopsy could also assess the possibility of recurrence in postoperative *KRAS* mutant CRC patients, in their ctDNA [2].

*RAS* mutations in CRC are of great significance, affecting tumor development, growth and resistance to chemotherapy [2]. Identifying *RAS* mutation status is necessary for all stage IV CRC patients, because only those with *RAS* wild-type status benefit from anti-EGFR treatment, according to the 2017 ASCO/AMP/CAP guidelines [7]. Additionally, there is evidence that RAS mutations have prognostic value [2]. Guo et al., showed that *KRAS* mutations are associated with shorter OS in stage IV CRC, whereas *NRAS* mutations are associated with shorter OS in stage I-II CRC [8]. Amongst *KRAS* mutations, those that involve mutations in codons 12 and 61 present worse prognosis [2]. On the other hand, codon 146 mutations are associated with better prognosis [2]. Ucar et al., demonstrated that multiple *KRAS* mutations were also correlated with better prognosis compared with single mutations [1]. Nonetheless, the actual role of *RAS* genes as prognostic markers remains questionable.

Many efforts have been made over the years to produce an effective RAS-targeted regimen; however, no such a regimen is available [2]. Recent, promising studies are ongoing, especially in *KRAS* G12C inhibitors [9]. Co-administration of the above with checkpoint blockers or other immunotherapies could potentially constitute the future therapeutic strategy for patients with RAS-mutant CRC [9].

To this end, we conducted a retrospective, multicenter study in which various *RAS* mutations were identified in stage I-IV CRC patients, these were correlated with epidemiological or tumor characteristics and their possible prognostic significance was evaluated.

## 2. Materials and Methods

### 2.1. Enrolled Patients

During the period 12/1998–03/2022, 578 patients with histologically confirmed stage I-IV CRC at diagnosis were included in this retrospective study, from four collaborating academic oncology units specializing in CRC. Inclusion criteria were: age >18 years old and any CRC stage at diagnosis with reported mutated *RAS* status, whereas exclusion criteria involved a second solid malignancy. All enrolled patients were characterized as metastatic (either de novo metastatic or with progressive disease) at the time of the analysis.

Formalin-fixed, paraffin-embedded (FFPE) tissues were received from all the patients. The study was approved by the Ethics and Scientific Committees of the University General Hospital of Heraklion (No: 12058/01-12-2005) and patients signed their written informed consent.

### 2.2. DNA Extraction and Molecular Analysis

A pathologist examined the samples to confirm the best area for dissection. An Eppendorf piezoelectric microdissector was used to isolate the cancer cells for samples with a content of <80%. The DNA extraction method used has been previously described [10]. *KRAS* and *NRAS* mutational analysis of exon 2 (codon 12 and 13), exon 3 (codon 61) and exon 4 (codon 146) were performed using Sanger sequencing analysis followed by nested PCR amplification. PCR assays were carried out in 10 μL (multiplex) and 20 μL (nested) reaction volumes containing 50 ng of genomic DNA, 1 × PCR buffer, 2.5 mmol/L MgCl_2_, 200 nmol/L of each primer, 200 µmol/L of each dNTP, 1.25 U of KAPA Taq HotStart (KAPA BIOSYSTEMS, SouthAfrica, Cape Town) and DEPC water. Samples were denatured with an initial hold of 96 °C for 12,005 s followed by 40 cycles of 15 s at 96 °C, 30 s at the annealing temperature and 15 s at 72 °C. The annealing temperature was 55 °C for the first multiplex PCR, except for the nested PCR for which we used an annealing temperature of 58 °C. Pairs of primers used to amplify specific exons are listed in Table 1.

For the Sanger sequencing reaction, PCR amplification products were purified using the PCR Clean-up Kit (Macherey-Nagel, Duren, Germany) according to the manufacturer’s protocol. An amount of 3 μL of the purified product was used for the sequencing reaction using the BigDye Terminator v3.1 Cycle Sequencing Kit (Applied Biosystems Inc., Fostercity, CA, USA).

## 3. Results

### 3.1. Patient’s Characteristics

The median age of the 578 patients enrolled was 66 years (range: 28–88 years). Most of them were males (60%) and had a good performance status (PS) (94.6%). Also, most of the patients were of stage IV (49.1%) on diagnosis and had left sided tumors (71.4%), mainly on sigmoid (36.3%). Most of the patients who were firstly diagnosed as early CRC received adjuvant treatment (47.4%). *KRAS* and *NRAS* mutations were detected in 93.2% and 6.8% of the patients, respectively. All patients were assessed for screening for *KRAS/NRAS/BRAF* and no co-mutations were detected. The patients’ characteristics and demographics are demonstrated in Table 2 and Table S1; *KRAS*-G12C-mutated only patients are described in Table 3 and Table S1.

### 3.2. RAS Mutations

Of the 578 enrolled patients, 539 were *KRAS* and 39 *NRAS* mutated. Regarding *KRAS* mutations, *KRAS* G12D and G12V were more frequently detected (33.1% and 21.2%, respectively) (Table 4 and Table S1), whereas Q61R and G12D were the most common *NRAS* mutations (1.6% and 1.4%, respectively) (Table 5 and Table S1).

### 3.3. KRAS and NRAS Mutations: Prognostic Value Evaluation

#### 3.3.1. Correlation with PFS

**Figure 1:**
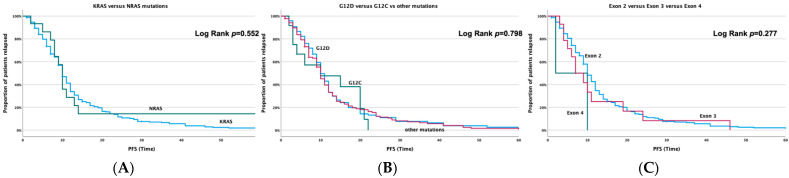
PFS regarding (**A**)*KRAS* and *NRAS* mutations; (**B**) *KRAS* G12D, G12C and other *KRAS* G12 mutations; (**C**) *KRAS* exon 2, exon 3 and exon 4 mutations.

Comparing PFS between the *KRAS-* and *NRAS*-mutated patients, no significant difference was observed (*p* = 0.552, Figure 1A). Concerning other *KRAS* mutations, PFS presented no statistical significance when *KRAS* G12D mutations were correlated with all the other *KRAS* G12 mutations. However, *KRAS* G12C mutations presented a worse PFS compared with others, however of no statistical significance (*p* = 0.798, Figure 1B). Although *KRAS*-exon-4-mutated patients seem to present a numerically better PFS, again no statistical significance was demonstrated when compared with other exons (*p* = 0.277, Figure 1C). Furthermore, no significant correlations were shown when the effect of age, performance status, treatment, tumor stage and tumor site were evaluated on PFS, and this is probably due to the small sample size in some patient groups.

#### 3.3.2. Correlation with Overall Survival

**Figure 2:**
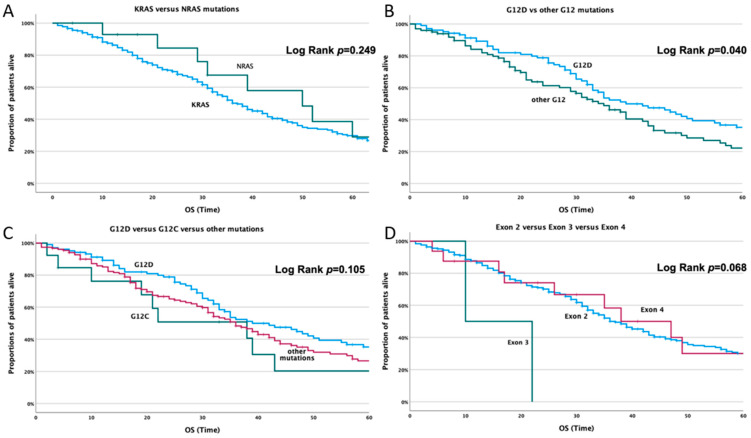
OS regarding (**A**) *KRAS-* and *NRAS*-mutated patients; (**B**) *KRAS* G12D and other *KRAS* G12 mutations; (**C**) *KRAS* G12D, G12C and other G12 mutations; (**D**) *KRAS* exon 2, exon 3 and exon 4 mutations.

There was no difference in overall survival (OS) between *KRAS-* and *NRAS*-mutated patients (*p* = 0.249, Figure 2A). *KRAS*-G12D-mutated patients had better prognosis compared with patients with other G12 mutations (*p* = 0.04, Figure 2B). They seem to maintain their better prognosis compared with G12C-mutated patients. On the other hand, G12C-mutated patients present a worse OS, however of no statistical significance (*p* = 0.105, Figure 2C). Comparing *KRAS*-exon-2-, 3- and 4-mutated patients, there was a trend for better OS for patients with *KRAS* exon 4 mutation (*p* = 0.068, Figure 2D).

## 4. Discussion

Although screening and treatment choices lead to a reduction in CRC prevalence, the disease still remains a major health issue [11]. Any knowledge on CRC tumorigenesis or metastatic mechanisms can reveal therapeutic options. Functional and/or structural changes in DNA can lead to these pathways [12,13]. On the way to the transformation from anormal colon epithelial cells to cancer cells, *KRAS* mutations occur, leading to the transformation from a small to a large adenoma [14]. Tumorigenesis, invasion and metastasis are promoted through extended proliferation via the *Ras*–*Raf*–MEK–ERK signaling pathway [15]. Also, immune reactions to cancer cells seem to be different in *KRAS*-mutated CRC patients [16].

*KRAS* mutations are detected in about 40% of CRC patients (stage II-IV) [17] and their role as negative predictive factors for the use of anti-EGFR therapy has been proven [18]. The same predictive value has the detection of *NRAS* mutation, at about 3–5% of CRC patients [17]. According to Hayama T, et al., the most common subtype of *KRAS* mutation was G12D (37.5%), followed by G12V (23%) and G13D (21.6%), as was demonstrated in a total of 200 patients; 37% of whom were *KRAS* mutated [19]. Similar results were shown by Bai B, et al. in a total of 135 patients [20]. In the current study, the most frequent mutations were G12D (33.1%), G12V (21.2%) and G13D (16.7%), and such results are in agreement with previous studies [19,20].

*KRAS* mutations have been proved as important predictive factors [21,22], but their prognostic significance is under evaluation. The worse prognosis of *KRAS*-mutated CRC patients has been shown in several studies [23,24,25]. Concerning our results, worse prognosis is related mainly to *KRAS* compared with *NRAS* mutations, and G12C mutations compared with other *KRAS* G12 mutations. He K, et al. have shown that *KRAS*-G12-mutated CRC patients with synchronous metastasis, have a phenotype of the disease that can lead to worse prognosis [26]. Regarding our results, comparing *KRAS* G12C and *KRAS* G12D mutations with other *KRAS* G12 mutations, it was demonstrated that *KRAS* G12C mutation seems to lead to a worse prognosis, regarding both PFS and OS. However, these results are not of a statistical significance, possibly because of the small number of patients carrying such mutations. On the contrary, *KRAS* G12D mutations presented a significantly better OS than other G12 mutations. *KRAS* G12C and G12D mutations are of great significance. *KRAS* G12C mutation is the first mutation that has been targeted therapeutically, whereas *KRAS* G12D mutation seems to be an inhibitory factor for an effective immune response to the tumor [27].

An important factor that has been poorly investigated in CRC is the presence of co-mutations. Studies in non-small cell lung cancer have shown distinct biologic behavior and prognosis in *KRAS/LKB1*-*, KRAS/TP53*- or *KRAS/p16*-mutated tumors [28]. In addition, our group has published previously the importance of LKB1 loss (assessed by immunohistochemistry) in the early stages of colon cancer and especially in *BRAFV600E*-mutated tumors [29]. One of the major weaknesses of the current study is the lack of NGS data for co-mutations and LKB1 protein expression. Moreover, given the retrospective nature of the current study, the sample size is relatively small; thus, presenting some limitations to demonstrate the full correlation between mutations and prognosis.

Several trials have attempted to find a therapeutic choice for *KRAS*-mutated CRC patients on preclinical and clinical setting. *KRAS* G12C has been the main target until now, including molecules like ARS-1620, AMG510, MRTX849, LY3499446 and JNJ-74699157 [30]. *KRAS* G12D has been used as a target for adoptive T-cell transfer, whereas a *KRAS* vaccine and molecules targeting the downstream or upstream pathway are under research as well [30].

It is common knowledge that molecular characterization of the tumor can lead to a better understanding of the biological course of the disease [26,31]. Taking for granted that personalized medicine is the main way of treatment nowadays, knowing the exact mutational status of CRC patients can lead to better treatment choices. According to previous research in combination with our results, detection of *KRAS* mutations, and especially G12C and G12D subtypes, are of great significance for CRC patients, have prognostic value and possible therapeutic implications. The current research underscores the need of a large database based on international collaboration in order to discuss these issues. Further research should be contacted to confirm this hypothesis.

## 5. Conclusions

In conclusion, to our knowledge this is the first study that makes an effort to detect the different subtypes of *RAS* mutations and examine their prognostic significance to evaluate the PFS and OS rates in CRC patients. Our results provide additional evidence for the prognostic significance of *RAS* mutations and especially *KRAS* generally and *KRAS* G12C and G12D mutations. Further studies remain to confirm these results and highlight their therapeutic implications.

## Figures and Tables

**Table 1:** Sequences of primer sets used for each mutation.

*KRAS*				
*KRAS*12_F	5’	ACTGGTGGAGTATTTGATAGTGTAT	3’	exon 2
*KRAS*12_EXR	5’	TGTATCAAAGAATGGTCCTGCAC	3’	
*KRAS*12_INR	5’	GGTCCTGCACCAGTAATATGC	3’	
*KRAS*61_EXF	5’	AGGTGCACTGTAATAATCCAGACT	3’	exon 3
*KRAS*61_INF	5’	TCCAGACTGTGTTTCTCCCT	3’	
*KRAS*61_R	5’	AACCCACCTATAATGGTGAATATCT	3’	
*KRAS*146_EXF	5’	CTCTGAAGATGTACCTATGGTCCT	3’	exon 4
*KRAS*146_INF	5’	AGACACAAAACAGGCTCAGGA	3’	
*KRAS*146_R	5’	GCCCTCTCAAGAGACAAAAACAT	3’	
*NRAS*				
*NRAS*12_F	5’	GGCTCGCCAATTAACCCTGA	3’	exon 2
*NRAS*12_EXR	5’	CACTGGGCCTCACCTCTATG	3’	
*NRAS*12_INR	5’	GCCTCACCTCTATGGTGGGAT	3’	
*NRAS*61_F	5’	ATTGAACTTCCCTCCCTCCCT	3’	exon 3
*NRAS*61_EXR	5’	ACCTGTAGAGGTTAATATCCGCAAA	3’	
*NRAS*61_INR	5’	ATTGATGGCAAATACACAGAGGA	3’	
*NRAS*146_EXF	5’	AGCGAGTAAAAGACTCGGATGA	3’	exon 4
*NRAS*146_INF	5’	TCGGATGATGTACCTATGGTGC	3’	
*NRAS*146_R	5’	TGGATCACATCTCTACCAGAGTTA	3’	

**Table 2:** Patients’ characteristics and demographics.

Characteristics	Frequency (N = 578)	%
Age median	66 (28–88 years)	
<70	354	61.4
≥70	224	38.9
Gender	578	
Male	347	60
Female	231	40
Performance status		
0–1	547	94.6
≥2	31	5.4
Stage at diagnosis		
I	5	0.9
II	62	10.7
III	227	39.3
IV	284	49.1
Location		
Cecum	77	13.4
Ascending	63	10.9
Transverse	24	4.2
Descending	34	5.8
Sigmoid	210	36.3
Rectum	169	29.3
Right/Left		
Right	165	28.6
Left	413	71.4
Adjuvant Treatment		
Yes	274	47.4
No	304	52.6
Adjuvant Regimen		
None	304	52.6
5FU-like	101	17.6
LOHP-based	175	29.8
First Line Regimen		
Irinotecan-based	308	53.3
LOHP-based *	246	42.5
5FU-based *	24	4.2
Metastasectomy		
Yes	95	16.5
No	483	83.5
*KRAS* mut	539	93.2
*NRAS* mut	39	6.8

* LOHP-based: trans-/-diaminocyclohexane-oxalatoplatinum-based treatment; 5FU-based: 5-fluorouracil-based treatment.

**Table 3:** Demographics of *KRAS*-G12C-mutated patients.

Characteristics	Frequency (N = 28)	%
Age	64 (28–83 years)	
<70	19	67.9
≥70	9	32.1
Gender		
Male	19	67.9
Female	9	32.1
Performance status		
0–1	27	96.4
≥2	1	3.6
Stage at diagnosis		
I–III	11	39.3
IV	17	60.7
Right/Left		
Right	6	21.4
Left	22	78.6
Metastasectomy		
Yes	10	37.7
No	18	64.3

**Table 4:** *KRAS* mutations.

*KRAS* Mutation	Frequency (N = 539)	%
G12D	190	33.1
G12V	121	21.2
G13D	96	16.7
G12C	28	4.8
G12S	27	4.7
G12A	21	3.6
A146T	15	2.6
A146A	6	1
A146V	6	1
Q61H	5	0.9
G12R	3	0.5
G13R	3	0.5
A59T	2	0.3
G13_V14 > D	2	0.3
G13C	2	0.3
K117N	2	0.3
Q61K	2	0.3
Q61L	2	0.3
Q61R	2	0.3
A146X	1	0.2
A59E	1	0.2
E62Q	1	0.2
G12S, G12V	1	0.2

**Table 5:** *NRAS* mutations.

*NRAS* Mutations	Frequency (N = 39)	%
Q61R	9	1.6
G12D	8	1.4
Q61K	7	1.2
G12A	3	0.5
G12V	2	0.3
G13R	2	0.3
Q61L	2	0.3
S145L	1	0.2
G12S	1	0.2
G13D, A59T	1	0.2
G13V	1	0.2
K117K	1	0.2
Q61H	1	0.2

## Data Availability

All relevant data are within the paper and its Supporting Information files.

## Supplementary Materials

The following supporting information can be downloaded at: https://www.mdpi.com/article/10.3390/cancers14143320/s1, Table S1: Raw data material.
